# Building Trust and Equity: The Role of the Native Hawaiian and Pacific Islander Workgroup in Shaping Inclusive Health Research within the CARE Registry

**DOI:** 10.1002/alz70860_104008

**Published:** 2025-12-23

**Authors:** Vaatausili Tofaeono, Christy Nishita, Justina P Tavana, Kekoa Lopez‐Paguyo, Van Ta Park, Josh D Grill, Janice Y. Tsoh, Poki'i Balaz

**Affiliations:** ^1^ American Samoa Community Cancer Coalition, Pago Pago, American Samoa; ^2^ Center on Aging, University of Hawaii, Honolulu, HI, USA; ^3^ Brigham Young University, Provo, UT, USA; ^4^ National Association of Pasfika Organizations, Springdale, AR, USA; ^5^ Asian American Research Center on Health (ARCH), University of California San Francisco, San Francisco, CA, USA; ^6^ University of California San Francisco School of Nursing, San Francisco, CA, USA; ^7^ The UC Irvine Institute for Memory Impairments and Neurological Disorders, Irvine, CA, USA; ^8^ University of California San Francisco School of Medicine, San Francisco, CA, USA; ^9^ Kokua Kalihi Valley Comprehensive Family Services, Honolulu, HI, USA

## Abstract

**Background:**

The Collaborative Approach for Asian Americans, Native Hawaiians, and Pacific Islanders Research and Education (CARE) Registry is a health research initiative aimed at addressing the underrepresentation of AANHPI populations in aging, Alzheimer's disease and related dementias, caregiving, and other health research. Guided by community‐based participatory research principles, CARE fosters trust and partnerships between academic and community stakeholders, enabling culturally tailored engagement strategies to advance recruitment, retention, and meaningful inclusion of AANHPI participants in research. Since October 2021, CARE has enrolled more than 10,000 AANHPI nationally and has referred over 5,000 to research studies, however some groups remain underrepresented.

**Method:**

Native Hawaiians and Pacific Islanders (NHPI) have a long history of colonization and research exploitation, which disrupted traditional knowledge systems, cultural practices, and sovereignty, leaving enduring impacts on their health and social structures. Research practices often mirrored these colonial dynamics, extracting data without proper representation, informed consent, or reciprocal benefits, fostering distrust within these communities. However, the premise of integrating NHPI epistemologies and Indigenous frameworks, such as the Samoan Talanoa process and the Two‐Eyed Seeing Approach, into Western research methods is to: reinforce community trust, emphasize the importance of cultural relevancy to improve health research participation, and underscore reciprocal research practices that blend Indigenous and Western ways of knowing in an inclusive environment.

**Result:**

The CARE model includes a Specific Aim to build trust and equity within NHPI communities. This led to the establishment of a NHPI workgroup under the CARE Community Advisory Board represents pivotal step toward decolonizing research paradigms and achieving the CARE registry's objectives in a culturally respectful and safe manner. This workgroup integrates NHPI epistemologies into the registry's design and implementation, aligning research methods with community values and lived experiences. By actively engaging NHPI stakeholders (e.g. caregivers, healthcare providers, investigators, etc.) in decision‐making, the group fosters trust, mutual respect, and shared power between researchers and community members, dismantling hegemonic practices that have historically marginalized Indigenous perspectives.

**Conclusion:**

This collaborative model prioritizes NHPI voices to enhance research participation and reduce health disparities but also paves the way for impactful, culturally informed research outcomes across the lifespan.

